# Extraction and immunomodulatory activity of the polysaccharide obtained from *Craterellus cornucopioides*

**DOI:** 10.3389/fnut.2022.1017431

**Published:** 2022-11-08

**Authors:** Caixuan Zhang, Ying Shu, Yang Li, Mingzhu Guo

**Affiliations:** College of Food Science and Technology, Hebei Agricultural University, Baoding, China

**Keywords:** *Craterellus cornucopioides*, polysaccharide, structural characterization, immunoregulation, pathway

## Abstract

In this study, we investigated the structural features of the polysaccharide obtained from *Craterellus cornucopioides* (CCP2) by high-performance liquid chromatography, Fourier transform infrared spectroscopy and ion chromatography. The results showed that CCP2 was a catenarian pyranose that principally comprised of mannose, galactose, glucose, and xylose in the ratio of 1.86: 1.57: 1.00: 1.14, with a molecular weight of 8.28 × 10^4^ Da. Moreover, the immunoregulation effect of CCP2 was evaluated both *in vitro* and *in vivo*. It displayed a remarkable immunological activity and activation in RAW264.7 cells by enhancing the phagocytosis of macrophages in a dose-dependent manner without showing cytotoxicity at the concentrations of 10–200 μg/mL *in vitro*. Additionally, Histopathological analysis indicated the protective function of CCP2 against immunosuppression induced by cyclophosphamide (Cy). Meanwhile, the intake of CCP2 had better immunoregulatory activity for immunosuppression BALB/c mice model. After prevention by CCP2, the spleen and thymus weight indexes of BALB/c mice model were significantly increased. The RT-qPCR and Western Blot results provided comprehensive evidence that the CCP2 could activate macrophages by enhancing the production of cytokines (IL-2, IL-6, and IL-8) and upregulating the protein expression of cell membrane receptor TLR4 and its downstream protein kinase (TRAF6, TRIF, and NF-κB p65) production of immunosuppressive mice through TLR4-NFκB p65 pathway. The results demonstrated that CCP2 could be a potential prebiotic and might provide meaningful information for further research on the immune mechanism.

## Introduction

The immune system comprises a heterogeneous population of cells that are relatively quiescent in a steady-state, however, they respond to inflammation, infection, and other perturbations (1). In clinic settings, patients with compromised immunity may be particularly vulnerable to normal and opportunistic infections (2). The innate immune system comprises innate dendritic cells, natural killers cells (NK), macrophages, mast cells (MCs) and NKT cells as primary defense entity (3, 4) and protects against invading pathogens in non-specific way (4, 5). One of the most important non-specific immune actions is phagocytosis, which is performed by macrophages (6). Macrophages are involved in antiviral, anti-tumor activities, hypersensitivity reactions, autoimmune diseases, and immune regulation in adaptive and innate immune responses *via* the production of cytokines (such as interferon γ), interleukin-6 (IL-6), interleukin-10 (IL-10), and tumor necrosis factor α (TNF-α) (7). After regulating the activation and inhibition of receptors, the immune system activates the pathogen associated pattern-recognition receptors (PRRs) (8–10).

Polysaccharides, as metabolic products of plants, animals, and microorganisms, have attracted considerable attention due to their therapeutic effects, and are considered the immunological molecules of the innate immune system (11). It enhances the ability of macrophages to resist external stress and survive under various conditions by promoting the integrity and stability of the outer membrane (12). Several studies have been conducted to investigate the pharmacological activities and active components of edible and medicinal plants (13–16). It showed that fungal polysaccharides are efficacious in the treatment of diabetes, hypolipidemia, oxidative stress, and obesity, as well as in the activation of innate immune cells and stimulation of cytokines secretions (17, 18).

Yu, et al. (19) demonstrated that the porphyra-derived oligosaccharides possessed antigen-specific immune responses by regulating the levels of IgG1, IgG2a, and OVA-specific IgE, and producing IL-2, IFN-γ, IL-4, and IL17 in ovalbumin (OVA)-sensitized mice. Wusiman, et al. (20) verified that the Lagenaria siceraria (Molina) standl polysaccharide and sulfated modified LSP50 could induce long-lasting and high hemagglutination (HI) titers, antigen-specific lgG-NDV antibody, splenic lymphocyte proliferation, high immune organ index, which could be served as a novel and effective vaccine adjuvant in chicken to induce specific immune responses against infections and diseases.

Therefore, the activations of macrophages induced by fungal polysaccharides are essential for the innate immune system.

Craterellus cornucopioides is wild, edible fungus, that is widely distributed around the world (China, Japan, Korea, North America, and Europe). In our previous studies (21–23), a natural immune heteroglycan (average molecular weight of 1.97 × 10^6^ Da) with the potential to activate RAW264.7 macrophages were obtained from *C. cornucopioides* (CCP) *in vitro*. This heteroglycan showed potent immunomodulatory properties and reversed immunosuppression by enhancing the development of the immune system and the activation of peritoneal macrophage phagocytosis *via* regulation of the TLR4-NFκB pathway in peritoneal macrophages of immunosuppressed mice, which shows excellent prospects for the commercial development of functional foods and medicines (21–24).

Similarly, polysaccharides obtained from *C. cornucopioides* (CCP2) also have strong immunoregulatory potential in the extrinsic pathway. However, to date, a comprehensive understanding of the immunomodulatory activity of CCP2 *in vitro* and its structural characteristics have not been reported. The structural and bioactivity diversities of CCP2 remain unclear. Generally, the comprehensive utilization of agricultural products has significant economic and social environmental benefits, and has thus gained growing interests in the development of agricultural products.

On this basis, the structure information and immunomodulatory activity of CCP2 were investigated by FTIR, and in terms of monosaccharide composition. The proliferation, phagocytosis, and morphology of RAW264.7 cells were applied to understand the relationship between structural properties and biological activities, which further expands the application and advantages of *C. cornucopioides*.

## Materials and methods

### Materials and reagents

The fruiting body of *C. cornucopioides* was collected at the Junzi mountain of Shizong in Yunnan Province, P.R. Different monosaccharide standards (L-rhamnose, D -glucose, D-mannose, D-galactose, D-arabinose, and D-xylose) and DEAE-52 column (1.6 cm × 100 cm) were provided by Solarbio Biological Technology Company (BJ, CHN). Neutral red and 3-(4,5-Dimethylthiazol-2-yl)-2,5-diphenyl-tetrazolium bromide (MTT) were provided by Sigma Company (St Louis, MO, USA). Phosphate buffered saline, dimethyl sulfoxide (DMSO), Dulbecco’s Modified Eagle Medium (DMEM), and fetal bovine serum (FBS) were purchased from Gibco BRL (NY, USA). All the other reagents were of analytical grade.

### Extraction and purification of CCP2

The *C. cornucopioides* powder was extracted with distilled water at 85°C for 2.5 h (twice) after degreasing with acetone. The water extract was concentrated and deproteinized using the sevag reagent [Chloroform: n-butanol = 4:1 (V:V), 30 min, 10 times]. Finally, three volumes of ethanol were added to precipitate the crude polysaccharide (CCCP), which were collected after centrifugation at 3,000 rpm at 25°C for 10 min and freeze-dried under −80°C after redissolving in water. The yield was calculated using formula 1 as follows:


(1) $$\mathrm{CCCP}\,\mathrm{yield}\%\,\frac{W\,C\,C\,C\,P\,\text{×}\,100}{W\,\text{sample}}$$

Where *W*_*CCCP*_ and *W*_*sample*_ are the weights of CCCP and *C. cornucopioides* powder, respectively.

CCCP (80 mg) was dissolved in distilled water (2 mL), purified by the DEAE-A52 column (1.6 cm × 100 cm), and eluted at the flow rate of 0.6 mL/min. The eluent contained a macromolecule that was discovered by HPLC and named *C. cornucopioides* polysaccharide (CCP2).

### Molecular weight of CCP2

The Mw of CCP2 was determined by high-performance gel permeation chromatography (Agilent-1200) with a Shodex OHpak gel SB-805HQ column (8.0 mm × 300 mm, 35°C) and a refractive index detector (30°C). The sample solution (20 μL, 5 mg/mL) was injected into the apparatus. Deionized water was used as the flowing phase at the flow rate of 0.6 mL/min. The standard curve was established using the T-series Dextran (T-2000, T-500, T-70, T-40, T-20, and T-10) (25, 26).

### Determination of monosaccharide composition of CCP2

The ICS2500 chromatography system (Thermo) with the high-performance anion chromatography column Carbo Pac PA20 (150 mm × 3 mm) and a dual pulse current sensor was used to determine the monosaccharide composition of CCP2 (NaOH at 2.00 and 10.00 mM was used as the eluent, the flow rate was 0.45 mL/min, and the temperature was set at 30°C). In total, 5.00 mg of CCP2 was hydrolyzed with 2 M Trifluoroacetic Acid (TFA, 5 mL) for 3 h at 120°C. Followed, the samples were diluted with ionized water according to the gradient. One milliliter CCP2 solution was injected into the apparatus. D-mannose, D-xylose, D-arabinose, L-rhamnose, D-galactose, and D-glucose were derivatized as standards.

### FT-IR analysis

The experimental methods were referred to the literature reports (27). Briefly, 1.00 mg of CCP2 and 150 mg of KBr were mixed evenly and pressed into flake. Pure KBr flake was used as the blank background, and then the polysaccharide sample was analyzed on an Fourier Transform Infrared Spectroscopy (FT-IR) spectrophotometer with a resolution of 4 cm^–1^ (range: 4000 –400 cm^–1^) (VECTOR 22, Bruker, Germany).

### Cell culture

The RAW264.7 cells were cultured in DMEM supplemented with 10% (v/v) FBS streptomycin (100 units/mL), and penicillin (100 units/mL) at 37°C, and 5% CO_2_ in a humidified atmosphere. Cells were passaged every 48 h for reserve.

### Cell phagocytosis assays

The experimental method was according to the literature reports (28). For the neutral red uptake assay, the cell suspension (5 × 10^4^ cell/mL) of the macrophages was added into 96-well plates at 37°C. After 4 h incubation, the supernates were removed and treated with different concentrations of CCP2 (0, 10, 25, 50, 100, 200, and 400 μg/mL) for 24, 36, and 48 h, respectively, and 100 μL of neutral red solutions were added and incubated for another 2 h. After staining, the cells were rinsed twice by Hank’s solution. Afterward, the cells were lysed with a lysis buffer [ethanol and 0.01% acetic acid at the ratio of 1:1 (100 μL per well) and detected at 540 nm].

### General observation

During the experiment, all BALB/c mice were carefully monitored daily for signs of disease, the body weight and water intake of mice were recorded daily. The feeding environment was as follows: temperature: 22 ± 0.5°C, humidity: 50 ± 5%, light–dark cycle: 12:12 h. The mental state, stool consistency, diarrhea and rectal bleeding were observed and recorded. The mice were fasted for 24 h after gavage and sacrificed on the 17th day.

### Histopathological observation

The experimental methods were referred to the literature reports (29).

### Establishment of cy-induced immunosuppressive BALB/c mice model and treatments

Protective effects of CCP2 on immunosuppression mice were evaluated using a cyclophosphamide (Cy)-induced immunocompromised model recommended by China Food and Drug Administration (CFDA Publication No. 107, revised 2012). Briefly, 50 mice were randomly assigned into 5 groups (*n* = 10) according to the double-blind experiment after a week of adaptive feeding, including normal control group (NCG, 0.9% NaCl), immunocompromised model group (Cy-induced, CyMG), and three CCP2 as preventive treatment groups [CCP2 + Cy (L, M, H)]: 100, 200, and 400 mg kg^–1^ day^–1^. The details were as follows:

(1) NCG: Mice were intragastric administration once daily with 0.9% normal saline (0.2 mL) for 17 consecutive days, and intraperitoneally injected administration with normal saline (0.1 mL day^–1^) at 10th day for 3 days.

(2) CyG: Mice were intragastric administration once daily with 0.9% normal saline (0.2 mL) for 17 consecutive days and intraperitoneally injected administration with Cy (0.1 mL day^–1^, Mw 261.09 Da) at 10th day for 3 days.

(3) [CCP2 + Cy (L, M, H)]: Mice were intragastric administration with once daily with CCP2 at the doses of 100, 200, and 400 mg kg^–1^ day^–1^, respectively, for 17 consecutive days and intragastric administration with Cy at 10th day for 3 days.

On the 18th day after the various treatments, the BALB/c mice in each group were killed through the cervical dislocation method. The spleen and thymus were dissected and weighed.

The organ index was calculated as follows:


(2) Spleen⁢index⁢(mg⁢per⁢ 10⁢g)⁢spleen⁢weight⁢(mg)body⁢weight⁢(g)⁢×⁢10

(3) Thymus⁢index⁢(mg⁢per⁢ 10⁢g)⁢thymus⁢weight⁢(mg)body⁢weight⁢(g)⁢×⁢10

### Western blot analysis

The experiment was conducted according to the method reported by Price et al. (30). Specifically, the total protein of peritoneal macrophage of BALB/c mice was extracted using radio immunoprecipitation assay lysis buffer according to the instruction of manufacture. After incubation of macrophages in 6-well plates (1 × 10^5^ cells/mL) for 36 h, the macrophages were used for the protein extraction. All the primary antibodies were diluted with PBS for 1000 times (Cell Signaling Technology, Danvers, MA, USA).

In brief, cell lysates were subjected to 10% SDS-PAGE and transferred to nitrocellulose NC membranes, and then incubated overnight at 4°C with anti-TLR4, anti-TRIF, anti-TRAF6, anti-P-NF-kB p65, and anti-GAPHD monoclonal antibodies after a 1 h blocking on (5% (w/v) non-fat milk. The membranes were subsequently washed with Tris Buffered Saline Tween (TBST) and incubated for 1 h at room temperature with corresponding secondary anti-bodies. Immunoreactive bands were detected using enhanced chemiluminescence (ECL) kit (Millipore Co., Billerica, MA, USA), GAPHD was used as internal control.

### Quantitative reverse transcription-polymerase chain reaction analysis

Quantitative reverse transcription Polymerase Chain Reaction (RT-qPCR) was conducted using SYBR RT-qPCR kit and Mx3000P™ RT-qPCR system (Stratagene, USA) in triplicate for each sample reaction according to previous report (31) to determine the mRNA expression of cytokines IL-2, IL-6, IL-8, and TNF-α. The total RNAs of peritoneal macrophage of BALB/c mice was extracted using Trizol reagent (Solarbio, Beijing, China) according to the instruction of manufacture and to synthesize cDNA by PrimeScript RT kit (Takara Biological Engineering Company, Dalian, China). The designed specific primers (Sangon Biotechnology company, Shanghai, China) were list in Table 1.

**TABLE 1:** Primers sequence of polymerase chain reaction (RT-qPCR).

Target gene	Forward primer	Reverse primer	Product size (bp)
IL-2	atgaacttggacctctgcgg	atgtgttgtcagagcccttt	129
IL-6	gatgaagggctgcttccaac	gcttctccacagccacaatg	128
TNF-α	ctgaacttcggggtgatcgg	tgctcctccacttggtggtt	157
IL-8	atgacttccaagctggccgtg	ttatgaattctcagccctcttca	302
GAPHD	agatccctccaaaatcaagtgg	ggcagagatgatgaccctttt	220

### Data analysis

In this study, all statistical analyses were performed using SPSS 20.0 software (SPSS, Inc., IL, USA). Data were expressed as mean ± standard error (SE). One-way analysis of variance (ANOVA) and *T*-tests were used to test for statistical significance. *P*-values less than 0.05 were considered statistically significant.

## Results and discussions

### Extraction, purification and purity of CCP2

**FIGURE 1:**
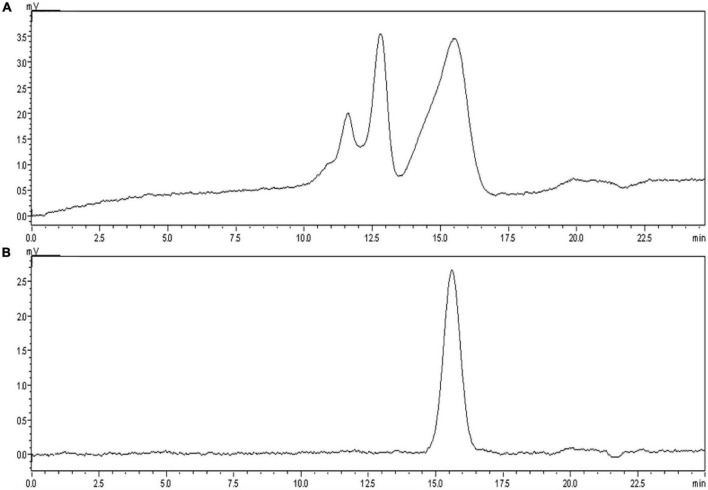
Weight average distribution of **(A)** CCP1 (crude polysaccharides eluted with 0.3 M NaCl) and **(B)** CCP2 (purified polysaccharides extracted from *C. cornucopioides*).

100 mg of CCCP was dissolved in distilled water (2 mL), purified by DEAE-A52 column (1.6 cm × 100 cm) and sequentially eluted with distilled water and 0.3 M NaCl at a flow rate of 0.6 mL/min. The eluant contained a macromolecule discovered by HPLC and named CCP1, which contained three fractions with similar polarity (Figure 1A). Further, the SephadexG-100 column (1.6 cm × 100 cm) was used to obtain CCP2. The single symmetrical peak (Figure 1B) at 15.592 min in HPLC indicates high purity. The UV absorption spectrum of thiirane revealed no obvious absorption peaks between 260 and 280 nm after full-wave scanning indicated little protein of CCP2. The average Mw of CCP2 was determined with a universal calibration curve using Dextran as a standard (32). Based on the calibration, the Mw of CCP2 was 8.28 × 10^4^ Da.

### The monosaccharide compositions and FTIR spectrum analysis of CCP2

**FIGURE 2:**
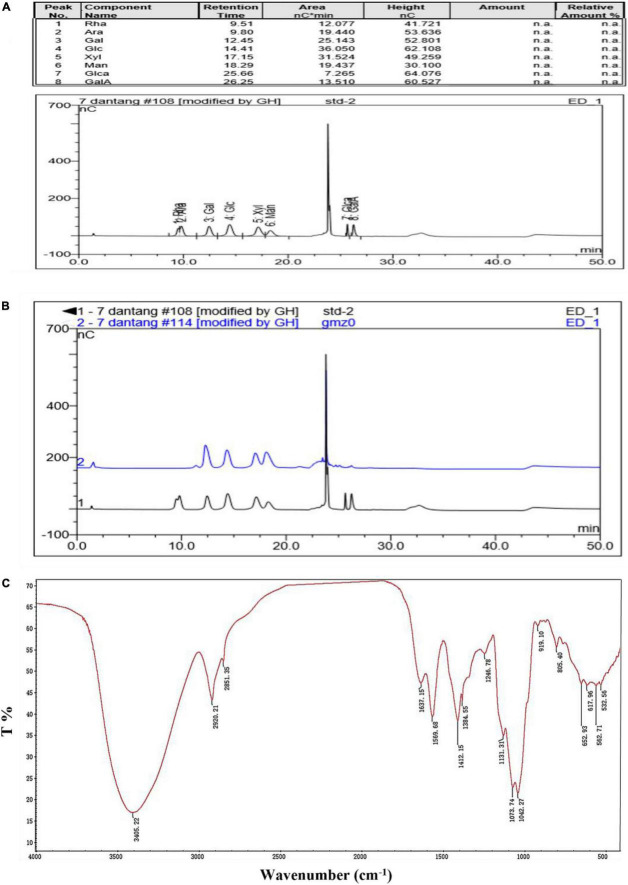
Monosaccharide composition [**(A)** Standards, **(B)** CCP2 (Blue line)] and the FTIR spectrum **(C)** of CCP2.

The retention time of monosaccharide standard (Figure 2A) and CCP2 after degrading by TFA acid (Figure 2B) were shown in the Figure 2 and Table 2. After comparing the remain time and area between standard and CCP2, the results indicated that the CCP2 composed of D-Mannose, D-Galactose, D-Glucose, and D-Xylose with the molar ratio of 1.86: 1.57: 1.00:1.14, showing mannose might be the backbone of the CCP2 chain (33).

**TABLE 2:** Monosaccharide composition of CCP2.

Component	Area (standard nC*min)	Area (CCP2 nC*min)	Molecular Weight (g/mol)	Molar ratio
Xyl	31.524	37.3365	150.13	1.14
Man	19.437	46.8032	180.16	1.86
Glc	36.050	44.0274	180.16	1.00
Gal	25.143	50.6455	180.16	1.57

nC*min is the unit of area provided by the gas chromatography (Figure 2A).

The absorption band of CCP2 was performed (range: 4000–400 cm^–1^). The band at 3405.22 cm^–1^ and 2920.21–2851.35 cm^–1^ were ascribed to the -OH and C-H stretching vibrations, respectively (21). The characteristic absorption peak of crystal water bending vibration was observed at 1637.15 cm^–1^ and the band at 1412.15 cm^–1^ was ascribed to -CH_2_ deformation absorption (34). Bands around 1246.78 cm^–1^ reflected the deformation vibration the of C-H bond. Similarly, bands between 1042.27–1073.74 cm^–1^ reflected the C-O-C stretching in the pyranose ring. The absorption characteristic peak at 919.10 cm^–1^ indicated β-type glycosidic bond. The peaks at 1131.31 cm^–1^, 1073.74 cm^–1^, and 1042.27 cm^–1^ indicated the existence of pyranoid ring structure (12).

### Effects of CCP2 on immunoregulation *in vitro*

#### CCP2 promoted phagocytosis activation of peritoneal macrophages

Recently, polysaccharides have been proven to participate in cell immune defense, proliferation, and differentiation (35). Several polysaccharides were used as immunotherapeutic agents in the treatment of cancer and were clinically applied in combination with chemotherapy. Phagocytosis is the most important index to evaluate the activation and function of macrophages (36–38). In this study, the neutral red uptake assay was used to evaluate the phagocytosis of macrophages *in vitro*.

**FIGURE 3:**
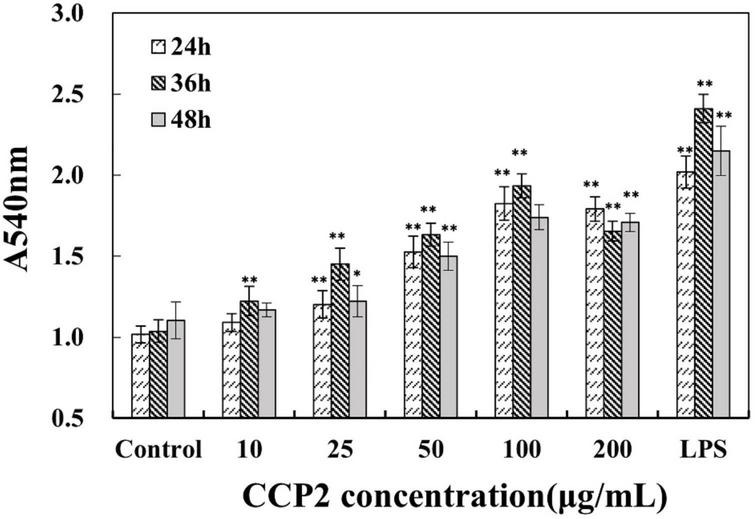
Effects of CCP2 on the phagocytic activity of macrophages. Cells were treated with CCP2 at various concentrations (10, 25, 50, 100, 200, and 400 μg/mL) for 24, 36, and 48 h, respectively. The control group was incubated with DMEM. *p < 0.05 and ^**^p < 0.01 were significant when compared to the Control group. Data were expressed as means ± SD.

Relative cell phagocytosis of macrophages in the presence of CCP2 was significantly increased in a dose-dependent manner (10–200 μg/mL) than that of the control group (*P* < 0.01), reached maximum value at 100 μg/mL (Figure 3). Meanwhile, CCP2 significantly enhanced the phagocytosis of macrophages in a time-dependent (24, 36, and 48 h) and reached maximum at 36 h. Generally, the phagocytosis of RAW264.7 was significantly increased, attaining a maximum value at 36 h and 100 μg/mL of CCP2 (*P* < 0.01). The above results indicated that CCP2 holded a strong potential to stimulate macrophages, which was the key participant in innate and adaptive immunity. Compared with previous reports, CCP2 showed stronger effect on the activation of the phagocytosis of macrophages (22, 34, 39).

### Effects of CCP2 on immunoregulation *in vivo*

#### The effect of CCP on thymus and spleen index of immunocompromised mice

The spleen and thymus index of various treatment groups were dissected and weighed accurately on the 17th day. The relative thymus index and spleen index of CyG group decreased significantly (6.94 ± 0.51 vs. 16.27 ± 0.89 mg/10 g, 20.11 ± 1.02 vs. 33.57 ± 1.32 mg/10 g) compared with NCG group (Table 3). To compared with the CyG group, all the indexes in CCP2 treatment groups remarkable increased. The result implicated that the organic damage and immune function of immunocompromised mice might be recovered after CCP2 treatments.

**TABLE 3:** The thymus and spleen index of immunocompromised mice.

	Group	Dosage (mg⋅kg^–1^⋅day^–1^)	Thymus index (mg 10 g^–1^)	Spleen index (mg 10 g^–1^)
	NCG	–	16.27 ± 0.89	33.57 ± 1.32
	CyG	–	6.94 ± 0.51^##^	20.11 ± 1.02^##^
Prevent	CCP2 + Cy (L)	100	9.46 ± 0.56**	22.97 ± 1.35**
	CCP2 + Cy (M)	200	13.32 ± 0.74**	29.47 ± 0.78**
	CCP2 + Cy (H)	400	11.27 ± 0.34**	26.38 ± 0.68**

Data are presented as mean ± SD, *n* = 6.

^##^*p* < 0.01 was significant when compared to the NCG group.

**p < 0.01 was significant when compared to the CyG group.

#### Effects of CCP2 on the spleen investigated *via* histological examinations in the BALB/c mice

The destruction of the immune system, lead to autoimmune diseases and inflammatory diseases, always accompanied the organic damage (40). As an vital extrinsic diagnostic technology, HE image is easily available to assess the immunosuppressive status of organism (41, 42).

**FIGURE 4:**
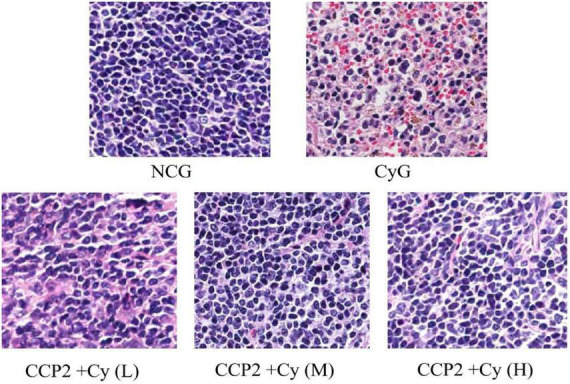
Effects of CCP2 on the spleen tissues showed in HE-stained histopathological images (scale bar = 100 μm, objective: 20×). NCG, normal control group; CyG, cyclophosphamide treatment group; CCP2 + Cy(L), Cy + CCP2 treatment group (100 mg kg^–1^ day^–1^); CCP2 + Cy(M): Cy + CCP2 treatment group (200 mg kg^–1^ day^–1^), CCP2 + Cy(H): Cy + CCP2 treatment group (400 mg kg^–1^ day^–1^).

In this study, the HE stained was performed to evaluate the effect of CCP2 on colon tissues ultrastructure. As shows in Figure 4, histological analysis showed that the ultrastructure of spleen cells in the NCG were dense and arrange regularly. To compared with NCG group, CyG group (B) showed unclear red and white pulp structure and obvious intercellular spaces dilatation. However, the administration of CCP2 could significantly decreased the extent of macroscopic and microscopic intestinal irregular arrangement of cells induced by Cy. As the ultrastructure of spleen cells in CCP2 + Cy (M) (200 mg kg^–1^ day^–1^) and CCP2 + Cy (H) (400 mg kg^–1^ day^–1^) groups were dense, arrange regularly with clear nuclei, red and white pulp structure, which were similar to the case of NCG group. Likewise, the microscopic structure of CCP2 + Cy (L) (100 mg kg^–1^ day^–1^) group also recovered slightly. The histopathological analysis showed that CCP2 could attenuate the immune lesions of spleen in immunosuppression mice after Cy intervention.

#### Effects of CCP2 on the secretion of cytokines of the immunosuppressive BALB/c mice

Cytokines are synthesized and secreted by immune cells (macrophages, monocytes, B and T cells, DCs and neutrophils, etc.) and non-immune cells (endothelial cells, epidermal and fibroblasts, etc.) after stimulation by immunogen, inflammatory factors, and exogenous stimulant with the biological activities on regulating inflammatory, innate or adaptive immune response (43). Such as TNF-α and IL-1β were released robustly by monocytes and macrophages after treated with LPS or Tripalmitoyl-S-glyceryl-cysteine (Pam3Cys, a lipopeptide). ECs granulocyte-monocyte-colony stimulating factor (GM-CSF), secreted granulocyte-colony stimulating factor (G-CSF), IL-6, IL-10, and IL-1α as major cytokines upon TLR stimulation (44, 45).

**FIGURE 5:**
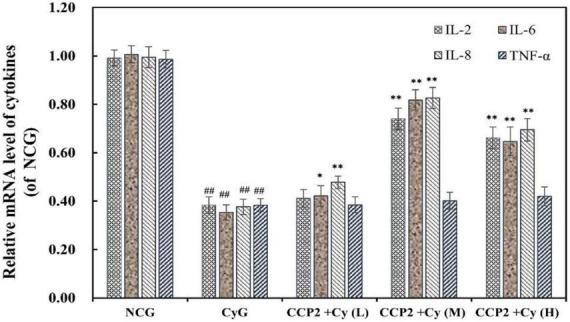
Effects of CCP2 on the secretion of cytokines (IL-2, IL-6, IL-8, and TNF-α) in the peritoneal macrophage of immunosuppressive mice induced by Cy. RT-qPCR analysis was relative to that of the reference gene (GAPHD). NCG: Normal control group, CyG: Cyclophosphamide treatment group, CCP2 + Cy (L): Cy + CCP2 treatment group (100 mg kg^–1^ day^–1^), CCP2 + Cy (M): Cy + CCP2 treatment group (200 mg kg^–1^ day^–1^), CCP2 + Cy (H): Cy + CCP2 treatment group (400 mg kg^–1^ day^–1^), ^##^*p* < 0.01 vs. the NCG group, *p < 0.05, ^**^p < 0.01 vs. the CyG group. Data were expressed as mean ± SD, *n* = 6.

To evaluate the immunosuppressive regulation capacity of CCP2, the mRNA expressions of immunological cytokines (IL-2, IL-6, TNF-α, and IL-8) in peritoneal macrophage of various treatment groups were analyzed (Figure 5). To compare with the NCG group, the expressions of IL-2, IL-6, IL-8, and TNF-α were suppressed significantly after Cy intervention (*p* < 0.01). As the comparison, CCP2 alleviated Cy-induced immunosuppression at a molecular level by promoting the production of IL-2, IL-6, and IL-8, but to different degrees in a dose-dependent manner. The CCP2 + Cy (M) and CCP2 + Cy (H) (200 and 400 mg kg^–1^ day^–1^) groups significantly enhanced the mRNA expression of above cytokines to compare with the CyG groups (*p* < 0.01) except TNF-α. Current data suggested that CCP2 capable of reversing the down-regulation of mRNA expressions to relieve the immunosuppressive.

#### Effects of *Craterellus cornucopioides* on protein expressions in abdominal macrophages in BALB/c mice

**FIGURE 6:**
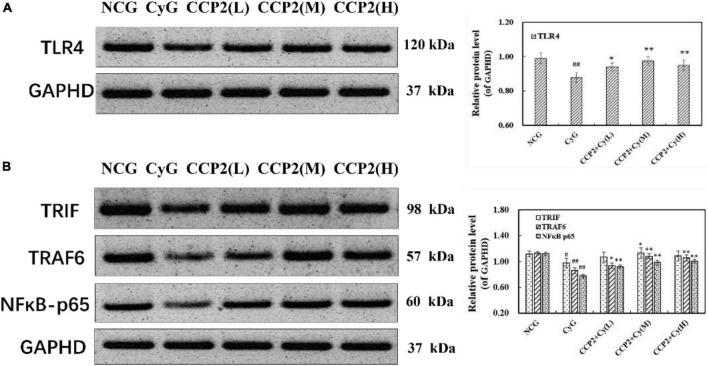
Detection of the protein expressions of TLR4, TRIF, TRAF6, and phosphor-NFκB-p65 by western blotting. **(A)** Effect of CCP on the expression of TLR4 in BALB/c mouse. **(B)** Effect of CCP on the expression of TRIF, TRAF6 and phosphor-NFκB-p65 in BALB/c mouse. GAPHD was used as an equal loading control. ^#^*p* < 0.05, ^##^*p* < 0.01 vs. the NCG group, *p < 0.05, *p < 0.01 vs. the CyG group. Data were expressed as means ± SD, *n* = 10.

Reportedly, TLRs and NF-κBs were involved in the stimulation of gene expression [such as Inducible Nitric Oxide Synthase (iNOS), IL-6, TNF-α mRNA] and cytokine secretion (such as NO, IL-6 and TNF-α) in immune responses. In view of the immunosuppression of Cy-induced injury as mentioned above, we elucidated an underlying mechanism of CCP2 effect *via* the TLR4-NF-κBp65 signal pathways, which were commonly involved in immune signaling cascades. The level of TLR4, TRIF, and TRAF6, and the phosphorylation of P- NFkB p65 were determined. As a result of Cy administration, the level of TLR4, TRIF, TRAF6, and P-NFκB p65 declined significantly, compared with those of mice in the NCG group. After administrating with CCP2, the phenomena (decrease of TLR4, TRIF, and TRAF6) were all alleviated significantly in a dose dependent manner, especially at the dose of 200 mg kg^–1^ day^–1^. Next, we evaluated the effect of CCP2 on the phosphorylation of p65. As shown in Figure 6, treatment with CCP2 increased the phosphorylation of p65 in a concentration-dependent manner. The CCP2 + Cy (M) and CCP2 + Cy (H) (200 and 400 mg kg^–1^ day^–1^) groups significantly enhanced the protein expression compared with the CyG groups (*p* < 0.01). Results suggested that CCP2 activated NF-κB signaling pathway which was implicated in transcriptional activation.

**FIGURE 7:**
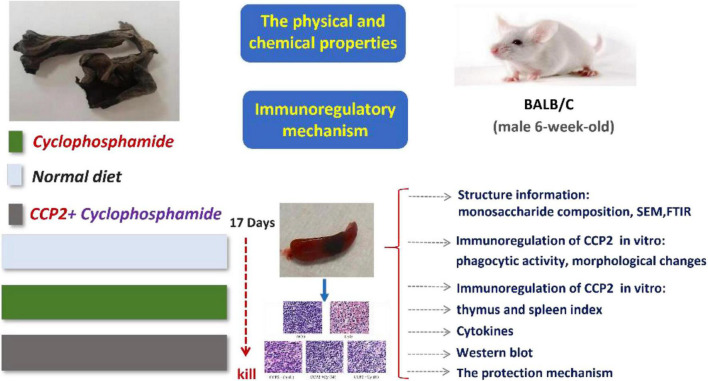
The sketch map of the immunoregulatory induced by CCP.

The main experiments contents and the sketch map was showed in Figure 7. In the light of the analysis conducted, we concluded that the receptor TLR4 plays a key role in the CCP2-modulated immunoregulation in immunosuppression mice model. Moreover, we showed that TLR4 in the pathogenesis of CCP2 modulated NF-κB pathways.

## Discussion

Macrofungus (mushroom) has been extensively applied as traditional oriental medicine and food component for centuries (46, 47). Several reports revealed the importance of Split gill mushroom [*Schizophyllum commune* (Fr.:Fr.)], Lingzhi (in China) [*Ganoderma lucidum* (W.Curt.:Fr.) P. Karst.], Shiitake mushrooms [*Lentinus edodes* (Berk.) Sing], among other (48, 49). Nowadays, mushrooms are used as natural product-based pharmaceuticals with higher treatment potential and lower toxic effects in different pathological processes.

Polysaccharides are made up of identical or different monosaccharides together with glycosidic linkages to be linear or branching structure, which have been produced as the first biopolymer on earth (50, 51). The macrofungus polysaccharides and polysaccharide complexes components attract considerable attention due to their bioactive [such as efficient immunomodulatory, anti-cancer (52) and anti-inflammatory effects (53, 54)]. However, the structural information of different functional polysaccharides needs to be analyzed to supply and to expand the application of macro-fungus polysaccharides. Thus, in this study, we obtained a polysaccharide that principally comprised of mannose, galactose, glucose, and xylose in the ratio of 1.86:1.57:1.00:1.14, obtained from *C. cornucopioides* (CCP2) that widely distributed around the world (China, Japan, Korea, North America and Europe).

There was a clear correlation between allowed conformations and linking pattern (55). As confirmed by reports, polysaccharides extracted from MAE showed excellent biological properties owing to their complete structure, functional glycosidic linkages with a higher Mw and uronic acid content. In this study, The CCP2 was a catenarian pyranose with the Mw of 8.28 × 10^4^ Da. The high structural diversity reflects the functional diversity of these molecules (55, 56). The number of structural factors such as monosaccharide composition, uronic acids content, molecular weight (Mw), glycosidic bond type in the backbone chain, and the esterification degree are profoundly affected on the antiradical, antioxidant, and antimicrobial activities of polysaccharides extracted from biological sources (5, 57).

Recently, the structure of numbers of different heteropolysaccharides had been precisely defined. It indicated that the heteropolysaccharides in mushrooms revealed prominently biological activities, in which β-D-glucan was mainly relative to immunomodulatory and anti-tumor activity. The most conversant polysaccharide in medicinal mushroom is β-glucan due to their ability on stimulating cytokine secretion ability of T cells, NK cells, and macrophage (proliferation and differentiation) (47, 58, 59). The water soluble β-glucan isolated from edible mushroom *Entoloma lividoalbum*, contains (1→3,6)-β-D-Glc*p*, (1→3)-β-D-Glc*p*, (1→6)-β-D-Glc*p*, and terminal β-D-Glc*p* glucosides, showed antioxidant and immune-stimulate activities on thymocyte, splenocyte, and macrophage (44). Based on the result of monosaccharide composition, CCP2 composed of mannose, glucose and galactose showed potential utilization in hypoimmunity population, might be a potential immunomodulatory.

In our previous study, we obtained a polysaccharide (CCP) with a molecular weight of 1.97 × 10^3^ kDa from edible *C. cornucopioides* fruiting bodies (21, 23). It was a heteroglycan with (1→3)-linked-β-D-Manp-(1→6)-linked-α-D-Galp backbone distributed by (1→4)-linked-α-D-Xylp-t-α-D-Manp and t-β-D-Glup units at O-6 and composed of mannose (48.73%), galactose (17.37%), glucose (15.97%), and xylose (17.93%), and stimulated macrophage function, rising phagocytosis, and activated cell morphology of RAW264.7 cells by TLR4-NFκB pathway. In the present paper, we investigated the chemical structure and biological activity of another *C. cornucopioides* polysaccharide. This study reported the isolation, structure analysis, and immunoregulatory activity of CCP2. Similarly, the immunomodulatory capacity was also found in CCP2. indicating the efficacy. Further studies including clinical trials need to be carried out to ascertain the safety of these compounds as adequate alternatives to conventional medicine. Our results showed that CCP2 could promote the phagocytosis of RAW264.7 cells in a concentration-dependent potency manner.

Cy has wide-spreading side-effects, such as hepatotoxicity and nephrotoxicity (60, 61). According to our results of thymus and spleen index (Table 2), and histological examination on the spleen of immunocompromised mice. We hypothesized that the immunosuppression in this group probably attributed to Cy side-effects on organic damage.

The stimulating factors affected adaptive immune cells (Th1, Th2, Th17, Tgd17, and CD8 T cells) to secrete IL-4, IL-5, IL-15, TNF-α, and chemokine CXCL8 (IL-8), which influenced neutrophils, macrophages (M1 and M2), and other granulocytes to fight against extracellular bacteria, tumors, viruses or extracellular parasites involved in immunologic processes of infection resistance, autoimmunity and allergic disease. It has previously been described that Cy polarizes the immune response from Th1 to Th2 (62). In the current study, mRNA and protein levels of all targeted elements were severely decreased in three CCP2-treatment immunosuppressed mice groups.

Some polysaccharides, characterized from plants, animals, fungi, etc., with various pharmacological properties by inducing cytokine secretion in immune cells, causing its segments similar to the cell membrane which were predominantly composed of various polysaccharides with species-specific monosaccharides or structures. The present data demonstrated that CCP2 significantly stimulated the mRNA expression of IL-2, IL-8, IL-6 to modulate immune response.

The western blot was used to explain the phenomenon. As an integral membrane protein in cytoplasmic domain, the toll protein is the first identified in D. melanogaster as potent classes of PRRs (63). It is an essential factor involve in the survival and development of the embryo along with patterning, and characterize in recognizing the polysaccharide structures of cell walls. Among TLRs, TLR4 is known to induce production of TNF-α and IL-6. In this study, the expression of cell surface receptor TLR4 was elevated significantly. Moreover, the production of TRIF, TRAF6, and phosphorylation of NF-κBp65 were detected after administration of CCP2, indicating the activation of TLR4- NF-kBp65 signaling pathway.

These results indicated the impact of Cy in suppressing immune system through diminishing immune cells production, circulation and infiltration. The chemotherapy might cause an overall depletion of adaptive immune system cells.

Nevertheless, the administration of CCP2 reversed the immunosuppression side-effects that caused by Cy, which provided us a better understanding of the molecular mechanisms of the activation of immune system. Further understanding of the signaling pathways might provide novel insights into the mechanisms of immunomodulation and new opportunities on rational application of CCP2.

## Conclusion

Polysaccharides obtained from fungi have attracted considerable attention due to their unique biological activities. In the present study, we investigated the chemical structure and immunoregulatory activity of CCP2 for the first time. Available data indicated CCP2 possessed immune-enhancing effect *in vivo* and *in vitro* to alleviate immunosuppression, which could be considered as a functional component of *C. cornucopioides* and an immunological modulator in the food nutrition industry.

## Data availability statement

The original contributions presented in this study are included in the article/supplementary material, further inquiries can be directed to the corresponding author/s.

## Ethics statement

This animal study was reviewed and approved by the Animal Ethical and Welfare Committee (AEWC).

## Author contributions

CZ designed and conceived the experiments. MG performed the experiments and wrote the manuscript. YS and YL contributed the reagents, materials, and analysis tools and analyzed the data. All authors have read and approved the final manuscript.
